# Clinical and surgical risk factors in the development of proliferative vitreoretinopathy following retinal detachment surgery: a systematic review protocol

**DOI:** 10.1186/s13643-016-0284-7

**Published:** 2016-07-08

**Authors:** Rishika Chaudhary, Janine Dretzke, Robert Scott, Ann Logan, Richard Blanch

**Affiliations:** Queen Elizabeth Hospital, Birmingham, UK; Neurotrauma Research Group, College of Medical and Dental Sciences, University of Birmingham, Birmingham, UK; Institute of Applied Health Research, University of Birmingham, Birmingham, UK; Moorfields Eye Hospital, Dubai, United Arab Emirates; Royal Centre for Defence Medicine, Birmingham, UK

**Keywords:** Proliferative vitreoretinopathy, Retinal detachment, Clinical risk factors, Surgical risk factors

## Abstract

**Background:**

Proliferative vitreoretinopathy (PVR) is a known complication of retinal detachment surgery. It has been postulated that the establishment of PVR involves inflammatory and ischaemic processes. Surgical and clinical risk factors contribute to making certain patients more vulnerable to developing PVR.

The objective of this systematic review is to identify and appraise the evidence on clinical and surgical risk factors and their utility in predicting the occurrence or worsening of PVR post-surgery.

**Methods:**

Electronic databases and grey literature will be searched dating from 1980. Studies will be eligible if they include patients that underwent retinal reattachment surgery for rhegmatogenous retinal detachment (RRD), with and without PVR, and where risk factors were measured before or during surgery. Screening, data extraction and quality assessment will be performed independently by two reviewers using pre-defined criteria. Should any models be identified, we will liaise with the Cochrane prognostic group to help define the most appropriate quality assessment criteria based on the PROBLAST tool which is in development. All findings will be tabulated and narratively synthesised. Studies presenting models or adjusted data will likely be more informative than studies reporting unadjusted results for a single risk factor. When clinically and methodologically appropriate, random effects meta-analysis will be performed.

**Discussion:**

This review will systematically and comprehensively retrieve evidence to evaluate the clinical and surgical risk factors associated with PVR. The identified evidence may aid standardisation of clinical practice and more effective management for improving patient outcomes following RRD surgery and will provide a clear reference point for vitreoretinal surgeons.

**Systematic review registration:**

PROSPERO CRD42016035848

**Electronic supplementary material:**

The online version of this article (doi:10.1186/s13643-016-0284-7) contains supplementary material, which is available to authorized users.

## Background

Proliferative vitreoretinopathy (PVR) occurs in 5–10 % rhegmatogenous retinal detachment (RRD) cases and is the main cause of surgical failure [1]. PVR occurs in a series of phases starting from the moment a retinal tear occurs and ending by apoptosis and contraction of membranes. Tissue fibrosis is characterised by the accumulation of an excessive number of fibroblasts followed by the unregulated deposition of collagen and other matrix components. The constant exposure to growth factors and cytokines causes cells to produce excessive extracellular matrix (ECM) components. A major component of PVR generation is from an exaggerated inflammatory reaction to retinal tears and detachment [2]. Retinal pigment epithelial (RPE) cells are released through retinal breaks and migrate along the retinal surface. RPE cells go through the epithelial-mesenchymal transition to form fibroblast-like cells that significantly contribute to scar formation by ECM production in the retina and therefore PVR. The unbalanced action of growth factors and cytokines is thought to be a causative element of fibrosis.

Classification of PVR in clinical practice and preclinical research is based on the ophthalmoscopic appearance, the subjective evaluation of the amount of membrane contraction and its distribution [3]. The classification is not related to the evolution of PVR. PVR becomes clinically significant if it predisposes a RD case to surgical failure, causing a re-detachment or limiting visual recovery post-operatively.

Considering the pathobiology of PVR, the majority of clinical factors associated with an accentuated healing response sufficient to cause PVR appear to include an inflammatory component or a release of RPE cells into the vitreous. Several studies have focused on the identification of a large number of overlapping risk factors and associations for the development of PVR, some of which are more consistent than others with the known underlying pathophysiology. These encompass the presence of a posterior vitreous detachment; longer RD duration; greater physical extent of the detachment; associated vitreous haemorrhage; clinical signs of intraocular inflammation; a history of previous retinal or lens surgery; and increased retinal tear size, especially giant retinal tears. In cases of RRD presenting with pre-existing PVR [4–7], there is a significant risk of progression to further advanced PVR. Surgical factors associated with PVR development included extensive cryopexy and laser retinopexy, failure to close retinal breaks, peri-operative scleral perforation and peri-operative vitreous haemorrhage [5, 8, 9]. This information can be used in surgical planning so that manoeuvres that risk intraocular haemorrhage or increase post-operative inflammation are avoided. Examples of factors to consider would include the following: method of retinopexy; type of tamponade and timing of surgery. Other associated factors included previous crystalline lens removal resulting in aphakia [10] or pseudophakia, the use of intravitreal air, the use of vitrectomy and a greater number of operations required to flatten the retina.

A scoping search on Medical Literature Analysis and Retrieval System Online (MEDLINE) found no existing systematic reviews looking at clinical and surgical risk factors. The aim of this systematic review is to identify and appraise the evidence on the prognostic utility of clinical and surgical risk factors in predicting the occurrence of PVR.

Specific objectives are to identify whether development or worsening of PVR is associated with:

- Pre-existing PVR
- Extent of retinal detachment
- Vitreous haemorrhage
- Retinopexy
- Large retinal break
- Lens status
- Surgical factors

To ensure rigour, the protocol for this systematic review has been guided by the Preferred Reporting Items for Systematic Reviews and Meta-Analysis Protocols (PRISMA-P) checklist (Additional file 1). The protocol has been registered on PROSPERO (CRD42016035848).

## Methods

### Study criteria

#### Type

Any studies are included where the prognostic factor is measured in advance of the outcome. A retrospectively identified cohort where the risk factors were measured prior to or at surgery would be suitable for inclusion. Studies are eligible if they include patients that underwent retinal reattachment surgery for RRD, with and without PVR. Studies reporting any prognostic models including relevant risk factors will also be included. Abstracts will be included.

#### Participants

In all included studies, the population will consist of a cohort of patients that underwent retinal reattachment surgery for RRD and were followed up to determine whether PVR developed post-operatively or whether established PVR worsened. The two groups will be analysed separately. Surgery will include scleral buckling, pars plana vitrectomy and other related intra-operative procedures required for reattachment.

#### Risk factors

Studies have to report of at least one of the following: clinical risk factors, surgical risk factors or prediction model for developing PVR after retinal reattachment surgery. Studies are eligible if risk factors were recorded using clinical information collected before and during the surgical intervention.

The existence of pre-operative or established PVR suggests that the cellular, extracellular and chemical elements required for wound healing are present in a pro-inflammatory ‘soup’. A total protein level represents the sum of all detectable proteinaceous components in the vitreous. It provides information on the state of inflammation, breakdown of blood-retinal barrier (BRB) and the severity of wound healing.

Detachments extending over two quadrants (six clock hours) are more likely to develop PVR [6]. It has been suggested that a larger detachment is associated with a greater disruption of the BRB through ischaemic injury resulting in a greater influx of serum components into the vitreous cavity [6]. Larger detachments are associated with PVR, supporting earlier scheduling of smaller macula-off RDs for surgery, to minimise the spread of the detachment and the risk of PVR [11]. Furthermore, disruption of the BRB is exacerbated by the requirement of extensive surgery for the management of large RDs.

Intraocular haemorrhage is an overt sign of BRB failure with direct leakage of fibrin and pro-inflammatory growth factors into the eye. Any form of intraocular haemorrhage occurring pre-operatively, peri-operatively or post-operatively was associated with the generation of PVR. The association of vitreous haemorrhage with PVR could be due to the release of serum elements creating a rich pro-fibrotic environment [12].

Cryotherapy increases blood aqueous breakdown from the associated chorioretinal trauma, as well as promoting RPE migration. Cryotherapy causes a release of RPE cells throughout the ocular fluid, due to alteration in the protein matrix on electron microscopy [13, 14].

Eyes with horseshoe tears are more likely to develop PVR than those with atrophic holes [15]. Cryotherapy has been found to be a stimulating factor for post-operative PVR in RDs due to horseshoe tears with curled posterior edges or retinal tears 180° and over [16].

It has been suggested that the posterior lens capsule may protect the anterior uvea, the site of active transport, from mechanical and physical irritation by the vitreous gel in phakic eyes [10]. It is possible that the intact lens provides a physical barrier for transmission of inflammatory cytokines from the anterior chamber to the vitreous cavity.

#### Outcome measure

The development of post-operative PVR or worsening of existing PVR is based on clinical biomicroscopy and surgical outcomes. PVR will be defined as recurrent or persistent RD, tractional RD or a combination of tractional RRD. Pre-existing PVR will be defined as those participants that have PVR prior to initial surgery (refer to Additional file 2).

### Identification of studies

#### Databases

The following electronic databases will be searched: MEDLINE, MEDLINE In-Process, Cochrane Library, Excerpta Medica dataBASE (EMBASE) and Biosciences Information Service (BIOSIS) Citation Index. Search strategies will be adapted for individual databases. A sample strategy is shown in Additional file 3.

Searches for relevant literature on these databases will be done using a combination of free text and index terms related to ‘proliferative vitreoretinopathy’ and ‘tractional retinal detachment’, combined with terms related to risk factors, prediction and prognosis.

Searches will be restricted to human studies but without applying restrictions on language, publication type or date. Reference lists of included studies will be checked, and a citation search will be carried out to locate further articles citing the included studies.

#### Grey literature

Electronic Theses Online Service (EthOS) and conference abstract databases (Zetoc (British Library) and Conference Proceedings Citation Index (Web of Science)) will be searched electronically. Contact with experts will help find resources and reports that may not have been identified through routine searches of databases. In addition, searches of clinical trial registers such as UK Clinical Research Network (UKCRN) and UK Clinical Trials Gateway will be conducted. Main trial databases will be searched for any relevant ongoing studies (clinicaltrials.gov) [17].

After deletion of duplicates, titles and abstracts of the records retrieved from electronic databases will be screened to eliminate clearly irrelevant studies. Full-text articles will be obtained for the remaining records (i.e. those considered potentially relevant and those with unclear relevance requiring clarification) and assessed for eligibility based on the inclusion and exclusion criteria. Screening of studies will be performed independently by two reviewers. Any differences in opinions will be resolved through discussion until a consensus is reached. If necessary, a third person may be consulted. Reference management software will be used to facilitate the screening process and a PRISMA flow diagram used for documentation.

### Data extraction and coding

The following data will be collected from included studies using a standardised data extraction form:

- Details on study design
- Eligibility criteria for study participation and/or selection of study sample
- Exclusion criteria for the study
- Patient characteristics and features of retinal detachments
- Method of retinal reattachment surgery and related procedures
- Clinical risk factors investigated and method of detection/quantification
- Ascertainment of post-surgical PVR and length of follow-up
- Methods of analysis, including statistical tests and methods of variable selection, resultant regression equations and adjusted versus unadjusted results

Data extraction forms will be piloted on a sample of included studies to ensure that all the relevant information is captured and that resources are not wasted on extracting data that is not required. A second reviewer will check the data.

### Quality assessment

Two review authors will independently assess the risk of bias of the included studies using the Quality in Prognostic Studies (QUIPS) tool [18]. Important areas that will be considered when evaluating validity and bias in studies of prognostic factors include the following: participation, attrition, prognostic factor measurement, confounding measurement and account, outcome measurement and analysis and reporting. The quality assessment tool will be tailored to the review and piloted on a small selection of included studies. Quality assessment of studies may involve a degree of subjective judgement, and any differences in opinion will be resolved through discussion. Should any studies describing models be identified, the Probast steering group [19] will be contacted for the latest version of the (as yet unpublished) assessment tool.

### Data analysis and synthesis

Study findings will be tabulated and narratively summarised. Results will be grouped according to different clinical or surgical risk factors. The main summary outcome measure will be the relative risk of developing (or worsening of) PVR in the presence or absence of one or more surgical or clinical risk factor(s).

We will use thresholds for ordering of prognostic factor data as presented by the authors. If authors use more than one, results for all will be presented as statistical significance and direction of effect may depend on the threshold. Where findings are presented using a prognostic factor on a continuous scale, it may be possible to dichotomise using thresholds. A retinal detachment extending over two quadrants (six clock hours) would constitute as an extensive detachment. A large retinal break would be classed as greater than 1 disc diameter in size or a giant retinal tear (greater than three clock hours).

Assessment of clinical and methodological heterogeneity will determine the feasibility of meta-analysis. The main sources of heterogeneity are likely to be the type and timing of surgery. Where meta-analysis is considered feasible, random effects model is more likely to be appropriate given that there is likely to be between-study heterogeneity. Separate meta-analyses will be conducted for adjusted and unadjusted data and for different study designs. Statistical heterogeneity will be assessed using the *χ*^2^ test and the *I*^2^ statistic. Where sufficient data is available, the possibility of sub-group analysis will be considered. The main subgroups will relate to participant type, method of surgery (scleral buckling or vitrectomy surgery), timing of surgery and development or worsening of PVR.

Assessment of publication bias using funnel plots will be considered if there are sufficient studies (>10) in individual meta-analyses.

Where it is not appropriate to perform meta-analysis, included studies will be combined in a narrative synthesis, and where possible, the results of the included studies will be presented in a forest plot without a pooled estimate.

If a large number of relevant studies are identified, then a step-wise approach to analysis may be used, with studies reporting prognostic models and adjusted results likely to be more informative than studies reporting on a single prognostic factor or unadjusted results only. Scoping searches have found that there are unlikely to be any models.

The main study characteristics of any relevant ongoing research identified will be tabulated.

### Reporting

The review and its findings will be reported in accordance to the PRISMA guidelines. The findings will be discussed in the context of the strengths and weaknesses of both the review methods and the available evidence. This will include a discussion of the likely impact of any ongoing research on the review findings. The generalisability and implications of the review findings will be discussed in the context of the current and future clinical practice for the prevention of PVR.

## Discussion

Combined assessment of clinical and surgical risk factors may aid in better prediction of PVR risk following RD surgery. Awareness of these factors in the development of PVR may allow for a more careful planning of surgery to minimise risk. Pathways involved in the development of PVR could provide insight into future treatment targets.

This review will systematically and comprehensively retrieve evidence from a wide range of sources to identify evidence on clinical and surgical risk factors associated with PVR.

It is hoped that this review will provide a clear reference point for vitreoretinal surgeons and that the identified evidence will aid standardisation of clinical practice, with more effective management ultimately leading to improved outcomes for patients following RRD surgery.

## Abbreviations

BIOSIS, Biosciences Information Service; ECM, extracellular matrix; EMBASE, Excerpta Medica dataBASE; EthOS, Electronic Theses Online Service; MEDLINE, Medical Literature Analysis and Retrieval System Online; PRISMA, Preferred Reporting Items for Systematic Reviews and Meta-analyses; PVR, proliferative vitreoretinopathy; RRD, rhegmatogenous retinal detachment; UKCRN, UK Clinical Research Network

## Additional files

Additional file 1: PRISMA-P 2015 checklist. (81 kb) Additional file 2: Inclusion/exclusion criteria. (69 kb) Additional file 3: Sample search strategy for MEDLINE. (61 kb)
