# Supplementary material for: Clinical and surgical risk factors in the development of proliferative vitreoretinopathy following retinal detachment surgery: a systematic review protocol
Source: Syst Rev. 2016 Jul 8;5:107. doi: 10.1186/s13643-016-0284-7 (PMC4939038; doi:10.1186/s13643-016-0284-7)
Supplement: Additional file 2: — Inclusion/exclusion criteria. (69 kb) [file 13643_2016_284_MOESM2_ESM.docx]

***Table 1*** *Inclusion/exclusion criteria*

| **Study criteria** | **Inclusion** | **Exclusion** |  |  |
| --- | --- | --- | --- | --- |
| **Study design** | Any studies are included where the prognostic factor is measured in advance of the outcome. A retrospectively identified cohort where the risk factors were measured prior to or at surgery. | Retrospective studies |  |  |
| **Population** | Human studies. No age restrictions.  No PVR or established PVR pre-operatively | Animal studies.  Pre-existing ocular pathology other than PVR. | |  |
| **Study methodology** | Studies including patients undergoing retinal reattachment surgery (scleral buckling and pars plana vitrectomy for RRD | Studies that evaluate interventions against PVR without reference to the risk factors of PVR.  Studies comparing PDR against PVR. | | |
