# Supplementary material for: Clinical and surgical risk factors in the development of proliferative vitreoretinopathy following retinal detachment surgery: a systematic review protocol
Source: Syst Rev. 2016 Jul 8;5:107. doi: 10.1186/s13643-016-0284-7 (PMC4939038; doi:10.1186/s13643-016-0284-7)
Supplement: Additional file 3: — Sample search strategy for MEDLINE. (61 kb) [file 13643_2016_284_MOESM3_ESM.docx]

***Appendix 2*.**

Sample search strategy for MEDLINE

1. proliferative vitreoretinopathy.mp. or Vitreoretinopathy, Proliferative/

2. proliferative vitreo retinopathy.mp.

3. massive vitreous retraction.mp.

4. massive preretinal retraction.mp.

5. massive pre-retinal retraction.mp.

6. massive periretinal proliferation.mp.

7. massive peri-retinal proliferation.mp.

8. 1 or 2 or 3 or 4 or 5 or 6 or 7

9. predic*.mp

10. Clinical risk factors

11. Clinical marker

12. Prognos*

13. Surgical factors

14. Aphakic

15. Pseudophakic

16. Phakic

17. Retinopexy

18. Laser retinopexy

19. Cryotherapy

20. Vitreous haemorrhage

21. Pre-existing proliferative vitreoretinopathy

22. Established proliferative vitreoretinopathy

23. Pre-operative proliferative vitreoretinopathy

24. Detachment size

25. Extensive detachment

26. Large retinal break

27. 9 or 10 or 11 or 12 or 13 or 14 or 15 or 16 or 17 or 18 or 19 or 20 or 21 or 22 or 23 or 24 or 25 or 26

28. 8 and 27
